# Foreign Body Removed from the Urethra

**Published:** 1872-12

**Authors:** L. A. Harcourt


ART. III.—*Foreign Body Removed from the Urethra.* By L. A. Harcourt, M. D.

Mr. H., an American, aged 55 years, or thereabouts, the father of many children, having arrived at that mature age, if not at the age of discretion, without having gained any very definite knowledge of that mysterious channel extending from the neck of the bladder to the meatus urinarius, determined to satisfy his curiosity by exploring it for his own individual benefit and that of all future generations. The more he thought of it, the more insatiable his

Yankee curiosity became, until on the 15th July, 1872, he started on his voyage of discovery. Filled with the noble desire of adding one more link to the great chain of scientific discovery, jack-kinife in hand, he sought the woods, and selecting a young and tender bass-wood sprout, cut it to the required length, peeled and shaped it to his fancy. With this novel catheter, he began the work of exploration. Perseverance and sweet oil, they say, will accomplish anything. Mr. H. had no *oil*, but lots of perseverance, and so he kept on with the work until the instrument had reached the bladder. The friction necessary to introduce the twig generated considerable caloric, which soon dried up the moisture of the parts, and rendered the situation anything but comfortable. Like the fox in the pit, he then looked around for some means of escape from this unpleasant predicament, and concluded that the best plan would be to make the instrument retrace its course. With a view of imparting a retrograde motion to the twig, he began to make powerful traction, which, unfortunately for him, broke it about five or six inches from the meatus urinarius, leaving about three inches in the urethra, or in the urethra and bladder. For three days he suffered the most exquisite torture, until the pain became unindurable, and he was forced to look for surgical aid.

In this condition of affairs, my friend, Dr. Guinan, of New London, was called in. Having examined the case carefully and chloroformed the patient, he made an incision one-half or three-quarters of an inch in length into the urethra, about the junction of the spongy with the membraneous portion, through which he extracted the remainder of the sprout, to the great delight and comfort of his patient. The wound healed kindly, and in two or three weeks the patient was discharged entirely cured.

He is now willing to accept the testimony of other explorers, that there *is* an "open polar sea" in that direction, and has become a convert to the doctrine that "where ignorance is bliss 'tis folly to be wise."

